# Regulatory T cells and bioenergetics of peripheral blood mononuclear cells linked to pediatric obesity

**DOI:** 10.1097/IN9.0000000000000040

**Published:** 2024-04-25

**Authors:** Shannon Rose, Reid D. Landes, Kanan K. Vyas, Leanna Delhey, Sarah Blossom

**Affiliations:** 1 Department of Pediatrics, College of Medicine, University of Arkansas for Medical Sciences, Little Rock, AR, USA; 2 Arkansas Children’s Research Institute, Little Rock, AR, USA; 3 Department of Biostatistics, College of Medicine, University of Arkansas for Medical Sciences, Little Rock, AR, USA; 4 Department of Epidemiology, University of Michigan School of Public Health, Ann Arbor, MI, USA; 5 Department of Pharmaceutical Sciences, College of Pharmacy, University of New Mexico, Albuquerque, NM, USA

**Keywords:** adolescents, childhood obesity, glucose intolerance, glycolysis, immunometabolism, inflammation, insulin resistance, mTOR

## Abstract

**Background:**

Obesity-associated inflammation drives the development of insulin resistance and type 2 diabetes. We sought to identify associations of circulating regulatory T cells (Treg) with the degree of obesity (eg, body mass index *Z*-score [BMIz]), insulin resistance (homeostatic model of insulin resistance [HOMA-IR]), and glycemic control (HbA1c) in children and adolescents. We further sought to examine associations among bioenergetics of peripheral blood mononuclear cells (PBMCs) and CD4 T cells and BMIz, HOMA-IR, and HbA1c.

**Methods:**

A total of 65 children and adolescents between the ages 5 and 17 years were studied. HbA1c and fasting levels of plasma glucose and insulin were measured. We quantified circulating Tregs (CD3^+^CD4^+^CD25^+^CD127^-^FoxP3^+^) by flow cytometry, and measured mitochondrial respiration (oxygen consumption rate [OCR]) and glycolysis (extracellular acidification rate [ECAR]) in PBMCs and isolated CD4 T cells by Seahorse extracellular flux analysis.

**Results:**

Tregs (% CD4) are negatively associated with BMIz but positively associated with HOMA-IR. In PBMCs, OCR/ECAR (a ratio of mitochondrial respiration to glycolysis) is positively associated with BMIz but negatively associated with HbA1c.

**Conclusions:**

In children, Tregs decrease as body mass index increases; however, the metabolic stress and inflammation associated with insulin resistance may induce a compensatory increase in Tregs. The degree of obesity is also associated with a shift away from glycolysis in PBMCs but as HbA1c declines, metabolism shifts back toward glycolysis. Comprehensive metabolic assessment of the immune system is needed to better understand the implications immune cell metabolic alterations in the progression from a healthy insulin-sensitive state toward glucose intolerance in children.

**Trial registration:**

This observational study was registered at the ClinicalTrials.gov (NCT03960333, https://clinicaltrials.gov/study/NCT03960333?term=NCT03960333&rank=1).

## 1. Introduction

Approximately 20% of children and adolescents in the United States have obesity ^[1]^ putting them at an increased risk for type 2 diabetes (T2D), hypertension, and numerous cancers ^[2]^. Indeed, the incidence of T2D and cardiovascular dysfunction, conditions that were long considered adult-onset, is increasing among US adolescents ^[3]^. While there is a great need for effective preventative and treatment strategies to halt development of these serious life-long health conditions in children, we must first better understand the pathophysiology of obesity and its associated health complications in children.

The well-known link between obesity and systemic inflammation in adults seems to also apply to childhood obesity with increased levels of pro-inflammatory cytokines and adipokines as well as elevated C-reactive protein (CRP) measured in the blood of children with obesity as young as age 3 years ^[4]^. In a study of children aged 7 to 14 years, Codoner-Franch et al ^[5]^ classified children with obesity as metabolically healthy or at risk (defined as having at least 4 metabolic risk factors including abdominal adiposity, impaired glucose metabolism, low high-density lipoprotein [HDL] cholesterol, elevated triglycerides, and hypertension), and they reported that CRP, tumor necrosis factor α, and interleukin (IL) 6 were all increased in children with obesity regardless of metabolic risk factors. These findings suggest that inflammation may appear early in obesity before the onset of metabolic risk factors. Indeed, in a cohort of prepubertal children aged 5 to 9 years, we have found increased CRP in euglycemic overweight/obese children, regardless of insulin sensitivity, compared with normal-weight children ^[6,7]^.

Decreases in regulatory T (Treg) cells, a subset of CD4 T cells responsible for suppressing inflammation ^[8]^, have been reported in adults with obesity ^[9,10]^ and in adults with T2D ^[11]^. A critical role for Tregs in the development of insulin resistance has been demonstrated in mouse models of obesity. Adoptive transfer of CD4 T cells into lymphocyte-deficient obese mice reduced weight gain and improved glucose tolerance ^[12]^, while in situ induction of Tregs improved glucose tolerance and insulin sensitivity in obese wild-type mice on high-fat diet. Further, depletion of Tregs specifically in adipose tissue induced insulin resistance and increased inflammatory mediators in the adipose tissue ^[13]^ whereas in situ induction of Treg cells increased adipose resident Tregs and improved glucose tolerance in a high-fat diet obesity mouse model.

Targeting Treg cells, or alternatively T effectors, by modulating cellular energy metabolism may aid in reducing obesity-associated inflammation and preventing progression toward T2D and cardiovascular disease. T effectors (eg, T_H_1, T_H_2, T_H_17) produce more adenosine triphosphate (ATP) by aerobic glycolysis and express higher levels of the glucose transporter, Glut1, than Tregs, which primarily utilize mitochondrial oxidative phosphorylation, particularly fatty acid oxidation, to meet their energetic demands ^[14]^. Treg cells take up exogenous fatty acids (as opposed to high levels of glucose transported into T effectors), and differentiation into Tregs requires fatty acid oxidation while the differentiation into T effectors requires de novo fatty acid synthesis ^[15]^. Despite the potential for therapeutically targeting T cell metabolism ^[16]^, investigation of immune cell metabolism in the context of obesity or insulin resistance in children remains limited.

The purpose of this study was to identify associations of circulating Treg cells with the degree of obesity (eg, body mass index *Z*-score [BMIz]), insulin resistance (HOMA-IR), and glycemic control (HbA1c) in a pediatric cohort. We further sought to examine associations among bioenergetics of both PBMCs and isolated CD4 T cells and BMIz, HOMA-IR, and HbA1c.

Inflammation is considered a primary driver of insulin resistance in obesity. Targeting anti-inflammatory Treg cells and/or proinflammatory T effectors is a putative therapeutic avenue to combat obesity-associated inflammation. Our findings provide a better understanding of how Treg cells and bioenergetics of PBMCs relate to obesity and insulin resistance in childhood and support further metabolic profiling of the immune system in children to understand the implications of bioenergetic alterations of immune cell subsets in the progression toward insulin resistance and glucose intolerance.

## 2. Participants and methods

### 2.1 Participants

For this cross-sectional study, we recruited a cohort of 65 children and adolescents (5–17 years old) from a variety of sources targeting both healthy normal-weight children and children spanning the spectrum of obesity and metabolic health. Participants were recruited from the Center for Obesity and its Consequences in Health clinic and the Endocrinology and Diabetes clinic at Arkansas Children’s Hospital and by advertisements posted in the community via social media, local newsletters, and distribution of flyers through local fairs and events, daycare centers, schools, recreational centers, churches, stores, and other local health care providers. In addition, participants of previous research studies conducted at Arkansas Children’s who indicated willingness to be contacted for future research were also recruited. Exclusion criteria included body mass index (BMI) <5th percentile for age/sex, having an infection in prior 4 weeks, having a genetic or physical condition impacting mobility in past year, use of antipsychotics, thyroid hormone replacement therapy, inhaled or oral steroids, insulin, anabolic drugs, or stimulants, and having a diagnosis of type 1 diabetes, chronic lung disorders (except for well-controlled asthma), neurologic or developmental disorders (eg, epilepsy, developmental delay, or autism spectrum disorder), endocrine, hepatic, autoimmune, cardiac, or renal disorders.

#### 2.1.1 Ethical approval

This study was approved by the Institutional Review Board (IRB) at the University of Arkansas for Medical Science (IRB 228816, on December 6, 2018) and adhered to the Helsinki Declaration. The STrengthening the Reporting of OBservational studies in Epidemiology (STROBE) reporting guideline was followed. The parents or legal guardians of the study participants gave written informed consent and children older than 7 years of age gave assent to participate. Although not a clinical trial (eg, no intervention, no group assignment of subjects, and no blinding), this observational study was registered at the ClinicalTrials.gov (NCT03960333).

Confidentiality and anonymity: All participant data were handled with utmost confidentiality. Data were anonymized, and any personal identifiers were removed to ensure the privacy and confidentiality of the participants.

Publication consent: Participants were informed that their deidentified data may be used for publication purposes. They provided consent for the publication of study results in academic journals and presentations while ensuring their anonymity.

### 2.2 Study visit

Participants completed a single, early morning study visit taking place at the Pediatric Clinical Research Unit at Arkansas Children’s Hospital. Study visits occurred between May 2019 and January 2021. Participants arrived at the study site after an overnight fast, and after consent/assent and confirming inclusion/exclusion criteria, demographic (sex, age, race, and ethnicity) and anthropometric data were collected. Body mass was measured using a calibrated Avery Berkel HL122 Series Platform Scale (Dynamic Scales, Terre Haute, IN, USA) while height was measured using a stadiometer (Novel Products, Rockton, IL, USA). BMI was calculated and converted to BMI *Z*-scores according to CDC using https://cpeg-gcep.shinyapps.io/quickZ_CDC/. Following measurement of heart rate and systolic and diastolic blood pressure with a GE Carescape V100 Dinamap vital signs monitor, participants provided a fasting blood sample from which glucose, insulin, hemoglobin A1C (HbA1C) and CRP were measured (methods below). HOMA-IR was calculated from fasting insulin (µIU/mL) and glucose (mmol/L) using the following equation − HOMA-IR = (glucose × insulin)/22.5. These descriptive data summarizing the cohort are presented in Table 1.

**Table 1:** Summary of participant characteristics.

Variable	Mean ± SD	Median (25th, 75th)
Male	29 (45%)	
Age (years)	10.1 ± 3.0	10.3 (7.5, 12.2)
Weight (kg)	50.6 ± 21.3	48.0 (36.8, 59.2)
Height (cm)	144.9 ± 18.4	146.9 (130.6, 158.4)
Waist circumference (cm)	70.8 ± 16.9	70 (58, 81)
Hip circumference (cm)	81.4 ± 16.8	83 (69, 89)
Waist to hip ratio	0.87 ± 0.07	0.86 (0.82, 0.92)
BMIz	1.32 ± 1.22	1.32 (0.35, 2.12)
Systolic blood pressure (mmHg)	110.4 ± 12.9	111 (101, 118)
Diastolic blood pressure (mmHg)	65.2 ± 15.7	63 (58, 67)
Heart rate (bpm)	77.9 ± 12.8	78 (70, 88)
Glucose (mmol/L)	5.13 ± 0.55	5.06 (4.81, 5.36)
Insulin (mU/L)	13.73 ± 18.16	8.19 (4.91, 13.98)
HOMA-IR	3.15 ± 4.03	2.10 (1.12, 3.12)
HbA1c (%)*	5.4 ± 0.3	5.3 (5.1, 5.6)
CRP (mg/L)†	1.36 ± 1.95	0.31 (0.15, 2.21)

Unless noted, the sample size was *n* = 65.

* *n* = 64.

† *n* = 55.

BMIz, body mass index *Z*-score; CRP, C-reactive protein; HbA1c, glycemic control; HOMA-IR, homeostatic model of insulin resistance; SD, standard deviation.

#### 2.2.1 Blood collection and processing

Fasting blood was collected into ethylenediaminetetraacetic acid (EDTA) vacutainers that were gently inverted several times and placed on ice immediately after collection. Within 30 minutes of collection, blood was centrifuged at 1500 × *g* for 15 minutes at 4 °C to separate plasma, which was transferred into cryovials, flash frozen on dry ice, and stored at −80 °C for future analyses. Blood collected into serum tubes was allowed to clot for 30 to 60 minutes at room temperature prior to being centrifuged at 1200 × *g* for 15 minutes at room temperature to separate serum, which was transferred into cryovials, flash frozen on dry ice, and stored at −80 °C for future analyses.

#### 2.2.2 PBMC and CD4^+^ T cell isolation

PBMCs were isolated as previously described ^[6,17–20]^. Briefly, a volume of room temperature PBMC wash buffer (Ca^+2^/Mg^+2^ free dulbecco's phosphate-buffered saline (DPBS) with 0.1% bovine serum albumin (BSA) and 2 mM EDTA) equal to the volume of removed plasma was added to EDTA vacutainers. Diluted blood was gently mixed by inversion, and then layered onto an equal volume of Histopaque 1077 (Sigma, St. Louis, MO, USA) and centrifuged at 400 × *g* for 30 minutes at room temperature with no brake. The PBMC layer was transferred into a new conical tube containing PBMC wash buffer. PBMCs were washed 2 times and collected by centrifuging at 250 × *g* for 10 minutes. Contaminating red blood cells were lysed by briefly (~10 seconds) resuspending the PBMC pellet in 1 mL ice-cold water followed by 1:20 dilution with PBMC wash buffer. Viable PBMCs were counted using a hemacytometer and trypan blue exclusion. CD4 T cells were isolated from PBMCs by negative selection using antibody-labeled magnetic beads (CD4^+^ T Cell Isolation Kit, human, Miltenyi Biotec, Bergisch Gladbach, Germany) following the manufacturer’s protocol. Viable CD4 T cells were counted using a hemacytometer and trypan blue exclusion.

#### 2.2.3 Immunophenotyping

Freshly isolated PBMCs and CD4 T cells were incubated for 30 minutes at 4 °C with antibodies that bind to surface markers (anti-CD3, CD4, CD25, and CD127), followed by fixation/permeabilization with the FoxP3/Transcription Factor Staining Kit (eBioscience, San Diego, CA, USA) following manufacturer’s protocol, and a 30 minutes incubation at room temperature with the FoxP3 antibody. Antibodies used in this study are listed in Supplementary Table 1, http://links.lww.com/IN9/A1. Because we stained freshly isolated cells with viability >98% assessed immediately prior to staining, we did not include and live/dead probe. Samples were acquired on a Guava easyCyte 8HT (Millipore, Burlington, MA, USA) and data analyzed using FCS Express v7 (De Novo Software, Los Angeles, CA, USA). Tregs were defined as CD3^+^CD4^+^CD25^+^CD127^−^ FoxP3^+^^[21]^. The gating strategy is depicted in Supplementary Figure 1, http://links.lww.com/IN9/A1. Tregs are expressed as % CD4 T cells (eg, count of CD3^+^CD4^+^CD25^+^CD127^−^ FoxP3^+^ cells divided by the count of CD3^+^CD4^+^ cells multiplied by 100). We also measured the median fluorescence intensity (MFI) of FoxP3 staining in Tregs to evaluate the level of FoxP3 expression, which correlates with the functional suppressive capacity of Tregs ^[22]^.

#### 2.2.4 Extracellular flux (Seahorse) analysis

Freshly isolated PBMCs and CD4 T cells were plated on poly-d-lysine coated XF-96 well plates at a density of 250,000 cells per well in XF RPMI base (lacking phenol red, bicarbonate, glucose, pyruvate, or glutamine; Agilent 103576-100) freshly supplemented with 2 mM glutamine, 1 mM pyruvate, and 11 mM glucose. Final concentrations of inhibitors oligomycin, antimycin A, and rotenone were all 1 µM. Oxygen consumption rates (OCRs) were determined over 3-minute increments. Because cells were counted immediately before the assay and equal numbers of cells were plated per well, Seahorse data are internally normalized to cell count.

#### 2.2.5 Insulin, glucose, and CRP

Plasma insulin was analyzed by ELISA according to the manufacturer’s protocol (Human/Canine/Porcine Insulin Quantikine ELISA Kit, R&D Systems, Minneapolis, MN, USA). Plasma glucose and CRP were analyzed on a COBAS INTEGRA 400 plus analyzer (Roche, Indianapolis, IN, USA).

#### 2.2.6 Statistical analyses

For this work, we are primarily considering 3 factors: body mass measured with BMIz, insulin sensitivity measured with HOMA-IR, and HbA1c measured with HbA1C. We also give some consideration to inflammation measured with CRP. Rather than categorizing our cohort into groups based on arbitrary cutoff points for these parameters, we treat them as the continuous variables they are. However, we provide summary statistics and bivariate plots of these factors to understand how these factors are distributed and related to each other in this cohort.

We summarized the sample’s characteristics using percentages for categorical characteristics and either means (and standard deviations [SDs]) or medians (and interquartile ranges [IQRs], expressed as the 25th and 75th percentiles) for continuous characteristics, depending on the distributional nature of the continuous characteristic.

We used multiple linear regression to evaluate the relationship of BMIz, HOMA-IR, and HbA1c to each of the outcomes we collected; the regression model also included age and sex as terms. We report the coefficients and their 95% confidence intervals (CIs) for BMIz, HOMA-IR, and HbA1c. We considered coefficients statistically discernable if their 95% CIs did not include zero.

One participant had extreme data. Participant #41 had a HOMA-IR of 26.5, which was 1.67 times the next highest observed HOMA-IR. We performed the regressions with #41 in (the whole sample) and with #41 out (reduced sample). When one sample showed statistical significance on a covariate, and the other sample did not, we make note of whether the covariate’s estimate from the reduced sample was similar to the whole sample’s estimate. We decided a priori to refer to estimates as “similar” if the smaller estimate was at least 80% of the larger estimate.

Another participant, #82, had aberrant PBMC bioenergetics measures: the observed PBMC values were not physiologically possible. For this reason, we excluded #82 from the regressions of PBMC measures.

We also performed a correlation analysis to evaluate the relationship of CRP with each of the outcomes. We did not include CRP in the original regression because 10 participants were missing CRP values and adding CRP as an additional term in the regression model, along with the reduced sample size, would tend to overfit the regression model. In this correlation analysis, we computed the rank correlation of CRP with the residuals coming from the multiple linear regression on the outcome: the residuals account for the (linear) relationships of the outcome with BMIz, HOMA-IR, HbA1c, age, and sex.

We conducted the analyses in SAS/STAT version 9.4 (SAS, Inc., Cary, NC, USA). The data and SAS code are available upon request.

## 3. Results

**Figure 1.**
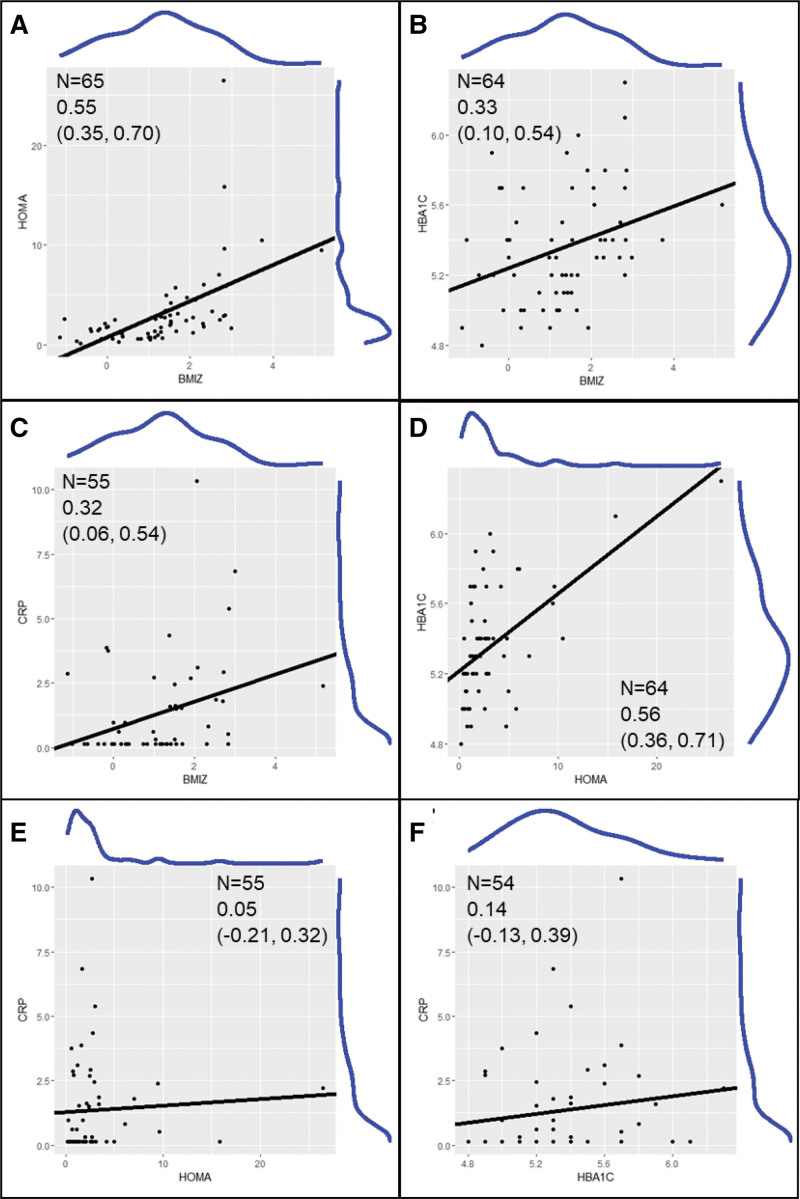
**Bivariate plots of BMIz, HOMA-IR, HbA1c, and CRP with best-fit lines (black lines) and empirical distributions (blue lines) on the margins.** Pearson correlations and their 95% confidence intervals (CIs) are also provided. BMIz, body mass index *Z*-score; CRP, C-reactive protein; HbA1c, glycemic control; HOMA-IR, homeostatic model of insulin resistance.

The summary statistics for the demographic and anthropometric measures and biochemical measures of metabolic health and inflammation of the entire cohort is presented in Table 1. Bivariate plots of each participant’s measures of BMIz, HOMA-IR, HbA1c, and CRP are presented in Figure 1 to demonstrate how these measures relate with each other; the marginal distributions of each measure are also provided to give insight into where most participants’ values fell and the spread of those values.

**Figure 2.**
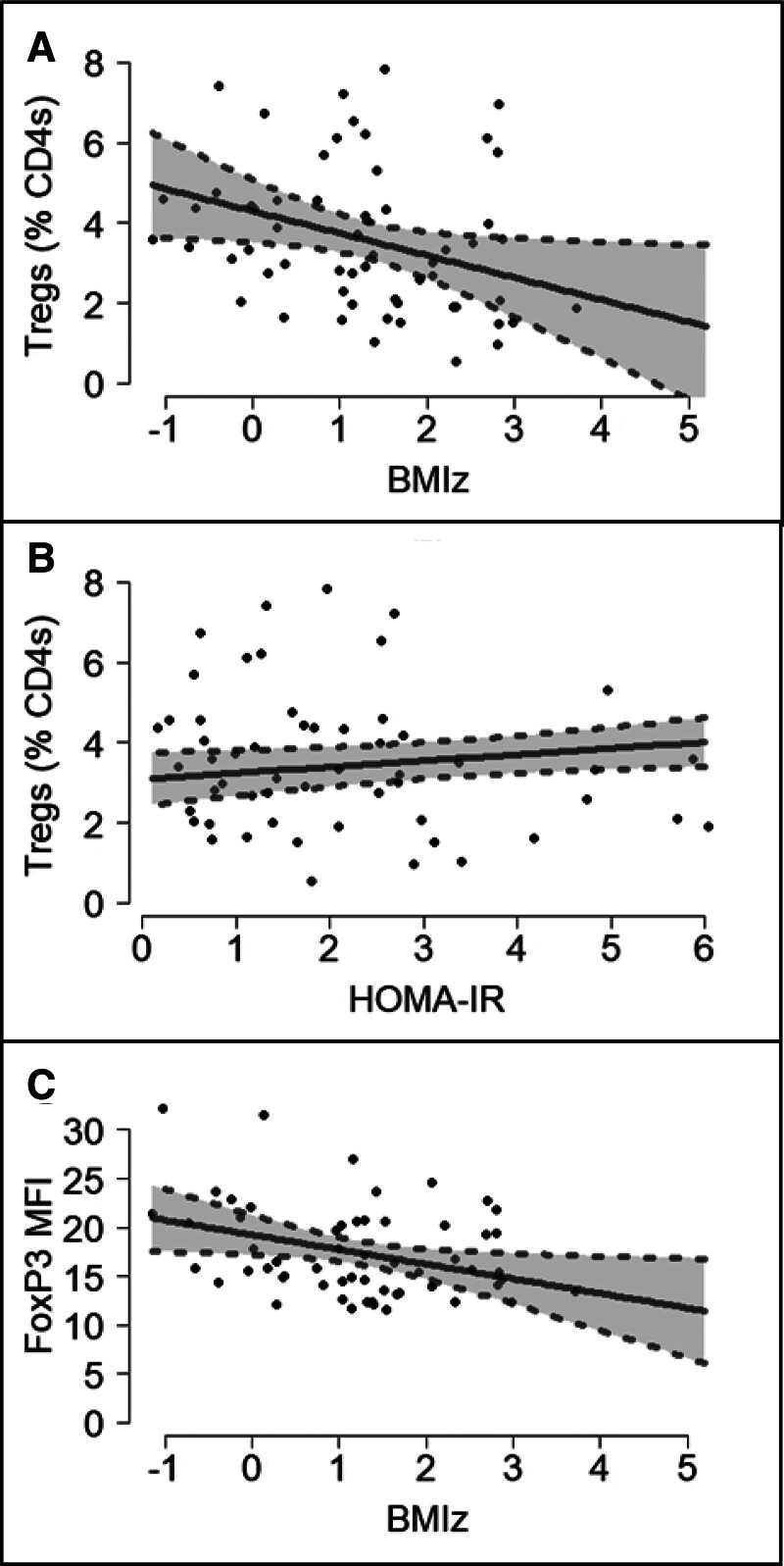
**Expected values and 95% confidence intervals, along with participants’ points, of Tregs (% CD4s) profiled by BMIz (A) and profiled by HOMA-IR (B), and FoxP3 MFI profiled by BMIz (C).** The expected values were computed at the average values of the other covariates in the model. BMIz, body mass index *Z*-score; HOMA-IR, homeostatic model of insulin resistance; MFI, median fluorescence intensity; Treg, regulatory T cell.

Table 2 presents the results of the multiple linear regressions for each outcome. Treg cells, expressed as a percentage of CD4 T cells, were found to be associated with both BMIz and HOMA-IR, but not HbA1c. Figure 2 demonstrates that while Tregs decreased with increasing BMIz, increases in HOMA-IR were associated with increases in Tregs. Further, when we examined the MFI of FoxP3 staining in Tregs, an indication of FoxP3 expression level, and a reflection of Treg function, we found FoxP3 MFI decreased as BMIz increased. When excluding extreme participant #41, the estimated coefficients of these 3 relationships were similar to those from the whole sample, but no longer discernably different from zero (Supplementary Table 2, http://links.lww.com/IN9/A1).

**Table 2:** Slopes for the 3 factors of interest estimated with multiple linear regression.

Outcome	Variable*	Slope	95% CI†	*P* value	RMSE‡	*R* ^2^	Model *P* value
Tregs (% CD4s)	HBA1C	−1.334	(−3.081 to 0.413)	0.131	1.74	0.16	0.098
	HOMA	**0.15**	**(0 to 0.3**)	**0.045**			
	BMIz	−**0.553**	(−**1.057 to** −**0.049**)	**0.032**			
FoxP3 MFI	HBA1C	−0.538	(−5.1 to 4.025)	0.814	4.54	0.15	0.117
	HOMA	0.24	(−0.16 to 0.63)	0.229			
	BMIz	−**1.499**	(−**2.821 to** −**0.177**)	**0.027**			
CD4 glycolysis-derived ATP production rate	HBA1C	8.538	(−16.01 to 33.086)	0.488	25.09	0.07	0.568
	HOMA	−0.76	(−2.96 to 1.43)	0.489			
	BMIz	−1.186	(−7.900 to 5.529)	0.725			
CD4 OXPHOS-derived ATP production rate	HBA1C	10.794	(−20.435 to 42.024)	0.491	31.92	0.04	0.848
	HOMA§	−1.52	(−4.31 to 1.27)	0.280			
	BMIz	3.917	(−4.625 to 12.458)	0.362			
CD4 % ATP-derived from glycolysis	HBA1C	0.892	(−4.707 to 6.491)	0.751	5.72	0.04	0.856
	HOMA	0.05	(−0.45 to 0.55)	0.847			
	BMIz	−0.748	(−2.279 to 0.784)	0.332			
PBMC OCR/ECAR∥	HBA1C	−**0.769**	(−**1.504 to** −**0.034**)	**0.041**	0.77	0.17	0.061
	HOMA	0.02	(−0.05 to 0.08)	0.636			
	BMIz	**0.255**	**(0.044 to 0.466**)	**0.019**			
PBMC basal respiration∥	HBA1C	1.512	(−10.517 to 13.541)	0.802	12.64	0.06	0.61
	HOMA	−0.67	(−1.77 to 0.42)	0.220			
	BMIZ	2.613	(−0.843 to 6.070)	0.135			
PBMC maximal respiration∥	HBA1C	−18.763	(−65.879 to 28.353)	0.428	49.5	0.06	0.674
	HOMA	−0.46	(−4.73 to 3.81)	0.830			
	BMIz	3.985	(−9.553 to 17.524)	0.558			
PBMC spare respiratory capacity∥	HBA1C	−20.275	(−59.099 to 18.549)	0.300	40.79	0.06	0.596
	HOMA	0.22	(−3.3 to 3.74)	0.903			
	BMIz	1.364	(−9.792 to 12.520)	0.807			
PBMC ATP-linked respiration∥	HBA1C	0.332	(−9.82 to 10.485)	0.948	10.67	0.05	0.696
	HOMA	−0.46	(−1.38 to 0.46)	0.319			
	BMIz	2.225	(−0.692 to 5.142)	0.132			
PBMC proton leak respiration∥	HBA1C	0.932	(−2.699 to 4.563)	0.609	3.81	0.09	0.42
	HOMA	−0.2	(−0.53 to 0.13)	0.230			
	BMIz	0.403	(−0.640 to 1.446)	0.442			

Associations significant at the 0.05 level are in bold font.

* The regression model also contained age and sex.

† Confidence intervals (CIs) that do not contain 0 indicate that the slope is significantly different from 0 at the 0.05 significance level.

‡ RMSE is the root mean square error from the regression; it may be interpreted as the standard deviation of the outcome after accounting for the variables in the regression model.

§ Significance observed in the reduced sample (excluding participant #41) but not in the whole sample.

∥ Excluded participant #82, as PBMC values were not physiologically possible.

BMIz, body mass index Z-score; ECAR, extracellular acidification rate; HbA1c, hemoglobin A1C; HOMA, homeostatic model of insulin resistance; MFI, median fluorescence intensity; OCR, oxygen consumption rate; OXPHOS, oxidative phosphorylation; PBMC, peripheral blood mononuclear cell; Treg, regulatory T cell.

In isolated CD4 T cells, we found that an increase in HOMA-IR was associated with a decrease in a surrogate marker of CD4 mitochondrial ATP production rate. However, this was only observed in the reduced sample excluding participant #41 with the extremely high HOMA-IR (Supplementary Table 2, http://links.lww.com/IN9/A1); the same coefficients estimated from the whole sample were in the same direction but attenuated (Table 2). BMIz or HbA1c was not found to be associated with CD4 ATP production by either the mitochondria or glycolysis.

**Figure 3.**
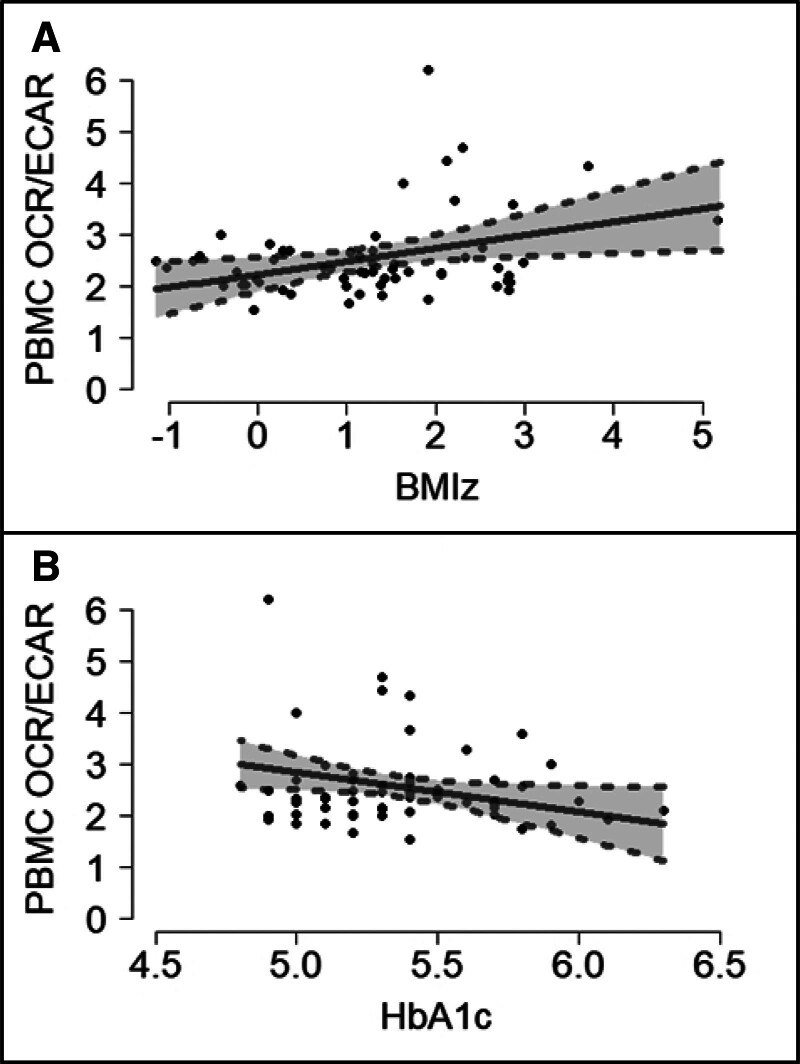
**Expected values and 95% confidence intervals, along with participants’ points, of PBMC OCR/ECAR profiled by BMIz (A) and profiled by HbA1c (B).** The expected values were computed at the average values of the other covariates in the model. BMIz, body mass index Z-score; ECAR, extracellular acidification rate; HbA1c, glycemic control; OCR, oxygen consumption rate; PBMC, peripheral blood mononuclear cell.

In PBMCs, we found that OCR/ECAR (a ratio of mitochondrial respiration to glycolysis) was associated with BMIz and HbA1c, but not HOMA-IR (Table 1). Figure 3 demonstrates that an increase in BMIz is associated with an increase in PBMC OCR/ECAR while an increase in HbA1c is associated with a decrease in PBMC OCR/ECAR. When excluding participant #41, the BMIz coefficient was attenuated and no longer discernably different from zero but the HbA1c association remained (Supplementary Table 2, http://links.lww.com/IN9/A1).

We evaluated whether CRP was associated with any of the outcomes after adjusting the outcomes for age, sex, BMI*z*, HOMA-IR, and HbA1c. There was no evidence of an association with CRP for any of the outcomes (Table 3).

**Table 3:** Spearman correlations (*r*) of CRP with residuals of the indicated outcome, after regressing the outcome on age, sex, BMIz, HOMA-IR, and HbA1c.

Outcome	*r*	95% CI	*P* value	*n*
Tregs (% CD4s)	0.09	(−0.20 to 0.36)	0.555	50
FoxP3 MFI	0.26	(−0.03 to 0.51)	0.074	49
CD4 glycolysis-derived ATP production rate	0.20	(−0.08 to 0.45)	0.157	51
CD4 OXPHOS-derived ATP production rate	−0.10	(−0.37 to 0.18)	0.479	51
CD4 % glycolysis-derived ATP production	0.21	(−0.08 to 0.46)	0.147	51
PBMC OCR/ECAR*	−0.20	(−0.45 to 0.08)	0.164	52
PBMC basal respiration*	−0.05	(−0.32 to 0.23)	0.740	52
PBMC maximal respiration*	−0.18	(−0.44 to 0.10)	0.191	52
PBMC spare respiratory capacity*	−0.18	(−0.44 to 0.10)	0.194	52
PBMC ATP-linked respiration*	−0.10	(−0.37 to 0.17)	0.462	52
PBMC proton leak respiration*	0.20	(−0.08 to 0.45)	0.158	52

* Excluded participant #82, as PBMC values were not physiologically possible.

CI, confidence interval; ECAR, extracellular acidification rate; MFI, median fluorescence intensity; OCR, oxygen consumption rate; OXPHOS, oxidative phosphorylation; PBMC, peripheral blood mononuclear cell; Treg, regulatory T cell.

## 4. Discussion and limitation

Inflammation is considered the primary driver of the progression from healthy obesity to insulin resistance, glucose intolerance, and eventually T2D ^[23–25]^. We previously reported evidence that T2D-driving inflammation is present very early, perhaps years before the onset of glucose intolerance. In a cohort of 5- to 9-year-old prepubertal children with overweight and obesity, we observed early insulin resistance driven by high insulin levels, as fasting glucose levels remained normal, and elevated markers of inflammation including CRP and the leptin/adiponectin ratio ^[6,17]^. This is consistent with National Health and Nutrition Examination Survey (NHANES) data showing elevated CRP in very young overweight/obese children, as young as age 3 years ^[4]^. Tregs, anti-inflammatory immune cells, are documented to be reduced in adults with obesity ^[9,10]^ and associated with the development of insulin resistance, type 2 diabetes, and fatty liver disease ^[26–28]^. Little is known regarding the status of Treg cells in children and adolescents with overweight and obesity.

In this study, we sought to determine how circulating Treg cells associate with BMIz, HOMA-IR, and HbA1C in children and adolescents spanning the spectrum of BMI, insulin sensitivity, and HbA1c. However, we took a decidedly different approach from what is often done when analyzing how outcomes relate to metabolic parameters: We treated the metabolic parameters on the continuum that they are, whereas, often, other studies dichotomize (or categorize) the metabolic parameters on some cutoff point. Such categorizations are a bit artificial or arbitrary (eg, someone right on the edge of a cutoff point for healthy weight/overweight is one meal or one night’s sleep away from changing categories). And such categorizations can reduce statistical power for detecting effects. Further, health can seldom be neatly categorized, rather health depends on a multitude of factors. Hence, we used multiple linear regression to evaluate the associations.

We report that BMIz is negatively associated with %Treg cells and FoxP3 staining intensity, while HOMA-IR is positively associated with %Treg cells. The inverse relationships of BMIz and HOMA-IR may be explained by a compensatory increase in Treg cells to combat inflammation associated with obesity and insulin resistance. The positive association between %Treg cells and HOMA-IR is consistent with findings from a recent study by Calcaterra et al ^[29]^ of a positive association between the Treg/T_H_17 ratio and HOMA-IR. Also consistent with our findings, Calcaterra et al did not observe any association between Treg/T_H_17 and HbA1c. In adults with T2D, reductions in both %Treg cells and the ratio of Tregs to T effector cells (Teff) were found, but neither HbA1c nor the duration of T2D were associated with %Tregs or Treg/Teff, suggesting that reductions in Treg cells occur early in the course of T2D development.

Due to the major role that cellular bioenergetics play in dictating immune cell fate and function, we also aimed to determine whether BMIz, HOMA-IR, and HbA1c were associated with mitochondrial respiration and glycolysis in CD4s and PBMCs. Mitochondrial respiration in CD4s was negatively associated with HOMA-IR, but because we did not observe this in the whole sample, only in the reduced sample excluding a participant with an extreme HOMA-IR, our confidence in this finding is reduced. In PBMCs, the ratio of mitochondrial respiration to glycolysis (OCR/ECAR) was positively associated with BMIz but negatively associated with HbA1c. We speculate that lymphocytes (the major component of PBMCs) may shift metabolism toward mitochondrial respiration under conditions of chronic overnutrition, but as glucose intolerance develops and glucose is chronically elevated, metabolism may shift toward glycolysis. Consistent with our data, Diaz-Morales et al ^[30]^ reported a decrease in mitochondrial respiration of PBMCs from adult T2D participants with an HbA1c greater than 6.5, but not in those with an A1C less than 6.5.

To begin to understand whether bioenergetics of immune cells is associated with inflammation, we tested for associations between each of the outcomes with CRP after adjusting for age, sex, BMIz, HOMA-IR, and HbA1c. As we were unable to obtain CRP measures on all participants, our power to detect these associations was limited. While none of the correlations of CRP with the outcomes were discernably different from zero, we note a correlation of 0.26 between Treg FoxP3 expression and CRP. Interestingly, Zeng et al ^[31]^, in their study of T2D adults, found a positive relationship between %Treg cells and the inflammatory cytokine, IL-6, and suggested that IL-6 may induce an upregulation of Treg cells.

Limitations of this study include the relatively small sample size and the focus on a single immune cell type. This study would have been strengthened by assessing other immune cell subsets (eg, Teff or T_H_17) and by assessing adipose resident immune cells. A larger sample size would have likely lessened the influence of participant #41’s measures on the results. Obtaining adipose tissue specimens in otherwise healthy children is not feasible. It is important to note that similarities between blood and adipose tissue T cell subsets have been reported by numerous groups ^[31–33]^, including mouse parabiosis studies demonstrating lymphocytes recirculate from adipose tissue back into blood.

Children and adolescents with overweight and obesity and those with T2D are more prone to severe infections (eg, flu and COVID-19) ^[34]^. Based on our findings that the bioenergetics and metabolism in immune cells in general (ie, PBMCs) and in CD4 T cells are related to excess weight, insulin resistance, and glucose tolerance, we speculate that impaired immunity in overweight/obese and T2D children may be related to metabolic adaptations to overnutrition and elevated blood glucose. A better understanding of the metabolic phenotypes across the entire immune landscape in children with overweight/obesity, insulin resistance, and impaired glucose tolerance is needed.

## 5. Conclusions

In this cohort of children and adolescents spanning the spectrum of obesity and insulin resistance, we report circulating Treg cells are negatively associated with BMIz, positively associated with insulin resistance but not associated with HbA1c. Metabolic stress and inflammation associated with insulin resistance may induce a compensatory increase in Tregs that occurs early, prior to changes in HbA1c. Among bioenergetic parameters of PBMCs, we found BMIz positively associates with PBMC OCR/ECAR while HbA1c negatively correlates with PBMC OCR/ECAR. Taken together our results indicate that at higher BMIs, metabolism of circulating PBMCs shifts away from glycolysis and toward mitochondrial respiration, but as glycemic control declines, metabolism shifts back toward glycolysis. A more comprehensive metabolic assessment of the immune system in children with obesity, particularly via longitudinal studies, is needed to better understand the role of immune cell metabolism in the progression from a healthy insulin-sensitive state toward glucose intolerance.

## Author contribution

SR, RDL, and SB designed the study, and SR obtained the funding. LD conducted participant recruitment and study visits and managed regulatory duties. SR and KKV conducted the experiments. SR and RDL analyzed data and wrote the manuscript. SR had primary responsibility for final content. All authors have read and approved the final manuscript.

## Conflicts of interest

The authors declare no conflict of interest, financial or otherwise.

## Funding

Research reported in this publication was supported by the National Institute of General Medical Sciences of the National Institutes of Health under Award Number 5P20GM109096 (Arkansas Children’s Research Institute, PI: Weber). Additional funds were provided by Arkansas Children’s Research Institute and Arkansas Biosciences Institute. The content is solely the responsibility of the authors and does not necessarily represent the official views of the National Institutes of Health or other funders.

## Acknowledgments

We thank all of the participants in the study. We acknowledge the technical assistance of Sirish Bennuri, MS, Alexandria Beebe, MS, Elisabeth Guy, RN, the Pediatric Clinical Research Unit (PCRU) team at Arkansas Children’s Hospital, and the Metabolism and Bioenergetics Core of the Center for Childhood Obesity Prevention at Arkansas Children’s Research Institute.

## Supplementary Material


