# Supplementary material for: Regulatory T cells and bioenergetics of peripheral blood mononuclear cells linked to pediatric obesity
Source: Immunometabolism (Cobham). 2024 Apr 25;6(2):e00040. doi: 10.1097/IN9.0000000000000040 (PMC11045398; doi:10.1097/IN9.0000000000000040)
Supplement: Supplementary file 1 [file in9-6-e00040-s001.pdf]

Supplementary Materials

Table S1. Antibodies used for Flow Cytometry.

Target	Fluor	Clone	Vendor	Cat No
**CD3**	FITC	UCHT1	Fisher	50 936 9
**CD4**	PerCP-Cy5.5	RPA-T4	Fisher	50 112 3693
**CD25**	PE	2A3	Fisher	BDB341009
**CD127**	PE-Cy7	eBioRDR5	Fisher	50 155 52
**FoxP3**	APC	236A/E7	Fisher	50 151 75

Table S2. Full statistics of muliple linear regressions.

SAMPLE	DEPENDENT	VARIABLE	EST	EST_SE	CI95	PVALUE	RMSE	RSQ	FVALUE	NumDF	DenDF	MODEL_PVALUE	TABLE2_ORDER	VARIABLE_ORDER	ORIG_ORDER
All in	Tregs (%CD4s)	MALE	-0.48	0.4628	(-1.41, 0.44)	0.301	1.740322	0.156752	1.970443	5	53	0.098201792	1	1	1
All in	Tregs (%CD4s)	AGE_YR	0.021	0.082967	(-0.146, 0.187)	0.804							1	2	2
All in	Tregs (%CD4s)	HBA1C	-1.334	0.870943	(-3.081, 0.413)	0.131							1	3	3
All in	Tregs (%CD4s)	HOMA	0.15	0.075129	(0, 0.3)	0.045							1	4	4
All in	Tregs (%CD4s)	BMIZ	-0.553	0.251419	(-1.057, -0.049)	0.032							1	5	5
All in	FoxP3 MFI	MALE	0.2	1.214364	(-2.24, 2.64)	0.87	4.54276	0.151906	1.862793	5	52	0.116938674	2	1	6
All in	FoxP3 MFI	AGE_YR	0.241	0.219424	(-0.199, 0.681)	0.277							2	2	7
All in	FoxP3 MFI	HBA1C	-0.538	2.273667	(-5.1, 4.025)	0.814							2	3	8
All in	FoxP3 MFI	HOMA	0.24	0.196776	(-0.16, 0.63)	0.229							2	4	9
All in	FoxP3 MFI	BMIZ	-1.499	0.658856	(-2.821, -0.177)	0.027							2	5	10
All in	CD4 Glycolysis-derived ATP Production Rat	MALE	11.04	6.727371	(-2.45, 24.54)	0.107	25.09327	0.068616	0.780914	5	53	0.567945675	7	1	31
All in	CD4 Glycolysis-derived ATP Production Rat	AGE_YR	-0.771	1.141589	(-3.061, 1.518)	0.502							7	2	32
All in	CD4 Glycolysis-derived ATP Production Rat	HBA1C	8.538	12.23879	(-16.01, 33.086)	0.488							7	3	33
All in	CD4 Glycolysis-derived ATP Production Rat	HOMA	-0.76	1.093942	(-2.96, 1.43)	0.489							7	4	34
All in	CD4 Glycolysis-derived ATP Production Rat	BMIZ	-1.186	3.347391	(-7.9, 5.529)	0.725							7	5	35
All in	CD4 OXPHOS-derived ATP Production Rate	MALE	5.94	8.558473	(-11.23, 23.11)	0.491	31.92333	0.036143	0.397479	5	53	0.848383649	8	1	36
All in	CD4 OXPHOS-derived ATP Production Rate	AGE_YR	0.04	1.452314	(-2.873, 2.953)	0.978							8	2	37
All in	CD4 OXPHOS-derived ATP Production Rate	HBA1C	10.794	15.57003	(-20.435, 42.024)	0.491							8	3	38
All in	CD4 OXPHOS-derived ATP Production Rate	HOMA	-1.52	1.391699	(-4.31, 1.27)	0.28							8	4	39
All in	CD4 OXPHOS-derived ATP Production Rate	BMIZ	3.917	4.258507	(-4.625, 12.458)	0.362							8	5	40
All in	CD4 %ATP derived from glycolysis	MALE	1.39	1.534475	(-1.68, 4.47)	0.367	5.723632	0.03511	0.385711	5	53	0.856404988	10	1	46
All in	CD4 %ATP derived from glycolysis	AGE_YR	-0.175	0.26039	(-0.698, 0.347)	0.504							10	2	47
All in	CD4 %ATP derived from glycolysis	HBA1C	0.892	2.791599	(-4.707, 6.491)	0.751							10	3	48
All in	CD4 %ATP derived from glycolysis	HOMA	0.05	0.249522	(-0.45, 0.55)	0.847							10	4	49
All in	CD4 %ATP derived from glycolysis	BMIZ	-0.748	0.763521	(-2.279, 0.784)	0.332							10	5	50
82 out	PBMC OCR/ECAR	MALE	0.16	0.203505	(-0.25, 0.57)	0.443	0.771838	0.173444	2.266266	5	54	0.060731429	11	1	51
82 out	PBMC OCR/ECAR	AGE_YR	0.014	0.034797	(-0.056, 0.084)	0.688							11	2	52
82 out	PBMC OCR/ECAR	HBA1C	-0.769	0.366449	(-1.504, -0.034)	0.041							11	3	53
82 out	PBMC OCR/ECAR	HOMA	0.02	0.033225	(-0.05, 0.08)	0.636							11	4	54
82 out	PBMC OCR/ECAR	BMIZ	0.255	0.105297	(0.044, 0.466)	0.019							11	5	55
82 out	PBMC Basal Respiration	MALE	-2.46	3.331971	(-9.14, 4.22)	0.463	12.63726	0.062634	0.721642	5	54	0.610077783	12	1	56
82 out	PBMC Basal Respiration	AGE_YR	0.689	0.569725	(-0.453, 1.831)	0.232							12	2	57
82 out	PBMC Basal Respiration	HBA1C	1.512	5.999846	(-10.517, 13.541)	0.802							12	3	58
82 out	PBMC Basal Respiration	HOMA	-0.67	0.543992	(-1.77, 0.42)	0.22							12	4	59
82 out	PBMC Basal Respiration	BMIZ	2.613	1.724029	(-0.843, 6.07)	0.135							12	5	60
82 out	PBMC Maximal Respiration	MALE	-1.01	13.05088	(-27.18, 25.15)	0.938	49.49844	0.055538	0.635081	5	54	0.673803446	13	1	61
82 out	PBMC Maximal Respiration	AGE_YR	3.564	2.231537	(-0.91, 8.038)	0.116							13	2	62
82 out	PBMC Maximal Respiration	HBA1C	-18.763	23.50059	(-65.879, 28.353)	0.428							13	3	63
82 out	PBMC Maximal Respiration	HOMA	-0.46	2.130742	(-4.73, 3.81)	0.83							13	4	64
82 out	PBMC Maximal Respiration	BMIZ	3.985	6.752789	(-9.553, 17.524)	0.558							13	5	65
82 out	PBMC Spare Respiratory Capacity	MALE	1.45	10.75401	(-20.12, 23.01)	0.894	40.78702	0.064231	0.741312	5	54	0.595915037	14	1	66
82 out	PBMC Spare Respiratory Capacity	AGE_YR	2.873	1.8388	(-0.813, 6.56)	0.124							14	2	67
82 out	PBMC Spare Respiratory Capacity	HBA1C	-20.275	19.36463	(-59.099, 18.549)	0.3							14	3	68
82 out	PBMC Spare Respiratory Capacity	HOMA	0.22	1.755745	(-3.3, 3.74)	0.903							14	4	69
82 out	PBMC Spare Respiratory Capacity	BMIZ	1.364	5.56434	(-9.792, 12.52)	0.807							14	5	70
82 out	PBMC ATP-linked Respiration	MALE	-0.63	2.812092	(-6.27, 5.01)	0.824	10.6655	0.053059	0.605147	5	54	0.696200314	15	1	71
82 out	PBMC ATP-linked Respiration	AGE_YR	0.597	0.480832	(-0.367, 1.561)	0.22							15	2	72
82 out	PBMC ATP-linked Respiration	HBA1C	0.332	5.063705	(-9.82, 10.485)	0.948							15	3	73
82 out	PBMC ATP-linked Respiration	HOMA	-0.46	0.459114	(-1.38, 0.46)	0.319							15	4	74
82 out	PBMC ATP-linked Respiration	BMIZ	2.225	1.455033	(-0.692, 5.142)	0.132							15	5	75
82 out	PBMC Proton Leak Respiration	MALE	-1.91	1.005718	(-3.92, 0.11)	0.063	3.814413	0.085571	1.010649	5	54	0.420490553	16	1	76
82 out	PBMC Proton Leak Respiration	AGE_YR	0.093	0.171965	(-0.252, 0.437)	0.592							16	2	77
82 out	PBMC Proton Leak Respiration	HBA1C	0.932	1.810985	(-2.699, 4.563)	0.609							16	3	78
82 out	PBMC Proton Leak Respiration	HOMA	-0.2	0.164198	(-0.53, 0.13)	0.23							16	4	79
82 out	PBMC Proton Leak Respiration	BMIZ	0.403	0.520379	(-0.64, 1.446)	0.442							16	5	80

SAMPLE	DEPENDENT	VARIABLE	EST	EST_SE	CI95	PVALUE	RMSE	RSQ	FVALUE	NumDF	DenDF	MODEL_PVALUE	TABLE2_ORDER	VARIABLE_ORDER	ORIG_ORDER
41 out	Tregs (%CD4s)	MALE	-0.47	0.467289	(-1.41, 0.46)	0.316	1.754293	0.137951	1.664285	5	52	0.159767235	1	1	81
41 out	Tregs (%CD4s)	AGE_YR	0.021	0.083637	(-0.147, 0.189)	0.803							1	2	82
41 out	Tregs (%CD4s)	HBA1C	-1.315	0.8792	(-3.08, 0.449)	0.141							1	3	83
41 out	Tregs (%CD4s)	HOMA	0.12	0.112597	(-0.11, 0.35)	0.288							1	4	84
41 out	Tregs (%CD4s)	BMIZ	-0.515	0.270984	(-1.059, 0.029)	0.063							1	5	85
41 out	FoxP3 MFI	MALE	0.23	1.225351	(-2.23, 2.69)	0.854	4.578028	0.142311	1.692425	5	51	0.153285716	2	1	86
41 out	FoxP3 MFI	AGE_YR	0.241	0.221127	(-0.203, 0.685)	0.281							2	2	87
41 out	FoxP3 MFI	HBA1C	-0.481	2.294762	(-5.088, 4.125)	0.835							2	3	88
41 out	FoxP3 MFI	HOMA	0.14	0.295491	(-0.45, 0.73)	0.635							2	4	89
41 out	FoxP3 MFI	BMIZ	-1.385	0.711228	(-2.813, 0.043)	0.057							2	5	90
41 out	CD4 Glycolysis-derived ATP Production Rat	MALE	11.85	6.807366	(-1.81, 25.51)	0.088	25.15413	0.081101	0.917894	5	52	0.476851003	7	1	111
41 out	CD4 Glycolysis-derived ATP Production Rat	AGE_YR	-0.787	1.144502	(-3.084, 1.51)	0.495							7	2	112
41 out	CD4 Glycolysis-derived ATP Production Rat	HBA1C	9.395	12.30868	(-15.304, 34.094)	0.449							7	3	113
41 out	CD4 Glycolysis-derived ATP Production Rat	HOMA	-1.85	1.667378	(-5.19, 1.5)	0.273							7	4	114
41 out	CD4 Glycolysis-derived ATP Production Rat	BMIZ	0.205	3.722602	(-7.265, 7.675)	0.956							7	5	115
41 out	CD4 OXPHOS-derived ATP Production Rate	MALE	8.12	8.436688	(-8.81, 25.05)	0.34	31.1747	0.095125	1.093302	5	52	0.375252642	8	1	116
41 out	CD4 OXPHOS-derived ATP Production Rate	AGE_YR	-0.003	1.418435	(-2.849, 2.844)	0.998							8	2	117
41 out	CD4 OXPHOS-derived ATP Production Rate	HBA1C	13.124	15.25472	(-17.487, 43.735)	0.394							8	3	118
41 out	CD4 OXPHOS-derived ATP Production Rate	HOMA	-4.46	2.06646	(-8.61, -0.32)	0.035							8	4	119
41 out	CD4 OXPHOS-derived ATP Production Rate	BMIZ	7.694	4.613595	(-1.563, 16.952)	0.101							8	5	120
41 out	CD4 %ATP derived from glycolysis	MALE	1.23	1.555094	(-1.89, 4.35)	0.432	5.74628	0.041581	0.451208	5	52	0.810479227	10	1	126
41 out	CD4 %ATP derived from glycolysis	AGE_YR	-0.172	0.261453	(-0.697, 0.352)	0.513							10	2	127
41 out	CD4 %ATP derived from glycolysis	HBA1C	0.719	2.811829	(-4.923, 6.361)	0.799							10	3	128
41 out	CD4 %ATP derived from glycolysis	HOMA	0.27	0.380901	(-0.5, 1.03)	0.486							10	4	129
41 out	CD4 %ATP derived from glycolysis	BMIZ	-1.029	0.850402	(-2.735, 0.678)	0.232							10	5	130
41 82 out	PBMC OCR/ECAR	MALE	0.13	0.205286	(-0.28, 0.55)	0.517	0.772767	0.182794	2.37102	5	53	0.051514285	11	1	131
41 82 out	PBMC OCR/ECAR	AGE_YR	0.014	0.034842	(-0.056, 0.083)	0.699							11	2	132
41 82 out	PBMC OCR/ECAR	HBA1C	-0.784	0.367237	(-1.52, -0.047)	0.037							11	3	133
41 82 out	PBMC OCR/ECAR	HOMA	0.05	0.050276	(-0.05, 0.15)	0.315							11	4	134
41 82 out	PBMC OCR/ECAR	BMIZ	0.208	0.116767	(-0.026, 0.442)	0.081							11	5	135
41 82 out	PBMC Basal Respiration	MALE	-2.61	3.384678	(-9.4, 4.18)	0.444	12.74106	0.053402	0.597991	5	53	0.701570493	12	1	136
41 82 out	PBMC Basal Respiration	AGE_YR	0.686	0.574463	(-0.466, 1.838)	0.238							12	2	137
41 82 out	PBMC Basal Respiration	HBA1C	1.419	6.054855	(-10.726, 13.563)	0.816							12	3	138
41 82 out	PBMC Basal Respiration	HOMA	-0.46	0.828936	(-2.12, 1.21)	0.585							12	4	139
41 82 out	PBMC Basal Respiration	BMIZ	2.322	1.925212	(-1.539, 6.184)	0.233							12	5	140
41 82 out	PBMC Maximal Respiration	MALE	-1.09	13.27254	(-27.71, 25.54)	0.935	49.96227	0.053905	0.603948	5	53	0.697103945	13	1	141
41 82 out	PBMC Maximal Respiration	AGE_YR	3.563	2.252677	(-0.955, 8.081)	0.12							13	2	142
41 82 out	PBMC Maximal Respiration	HBA1C	-18.81	23.74327	(-66.432, 28.813)	0.432							13	3	143
41 82 out	PBMC Maximal Respiration	HOMA	-0.35	3.250558	(-6.87, 6.17)	0.915							13	4	144
41 82 out	PBMC Maximal Respiration	BMIZ	3.84	7.54945	(-11.303, 18.982)	0.613							13	5	145
41 82 out	PBMC Spare Respiratory Capacity	MALE	1.52	10.93656	(-20.42, 23.45)	0.89	41.16884	0.064022	0.725051	5	53	0.607670395	14	1	146
41 82 out	PBMC Spare Respiratory Capacity	AGE_YR	2.875	1.856203	(-0.848, 6.598)	0.127							14	2	147
41 82 out	PBMC Spare Respiratory Capacity	HBA1C	-20.229	19.56442	(-59.47, 19.013)	0.306							14	3	148
41 82 out	PBMC Spare Respiratory Capacity	HOMA	0.11	2.678455	(-5.27, 5.48)	0.969							14	4	149
41 82 out	PBMC Spare Respiratory Capacity	BMIZ	1.51	6.220736	(-10.967, 13.988)	0.809							14	5	150
41 82 out	PBMC ATP-linked Respiration	MALE	-0.78	2.855148	(-6.5, 4.95)	0.787	10.74773	0.046125	0.512571	5	53	0.765521488	15	1	151
41 82 out	PBMC ATP-linked Respiration	AGE_YR	0.594	0.484589	(-0.378, 1.566)	0.225							15	2	152
41 82 out	PBMC ATP-linked Respiration	HBA1C	0.239	5.107578	(-10.006, 10.484)	0.963							15	3	153
41 82 out	PBMC ATP-linked Respiration	HOMA	-0.24	0.69925	(-1.64, 1.16)	0.731							15	4	154
41 82 out	PBMC ATP-linked Respiration	BMIZ	1.931	1.624014	(-1.326, 5.188)	0.24							15	5	155
41 82 out	PBMC Proton Leak Respiration	MALE	-1.92	1.022762	(-3.97, 0.13)	0.066	3.850017	0.079903	0.920525	5	53	0.475034967	16	1	156
41 82 out	PBMC Proton Leak Respiration	AGE_YR	0.092	0.173588	(-0.256, 0.441)	0.597							16	2	157
41 82 out	PBMC Proton Leak Respiration	HBA1C	0.926	1.82962	(-2.744, 4.595)	0.615							16	3	158
41 82 out	PBMC Proton Leak Respiration	HOMA	-0.19	0.250483	(-0.69, 0.32)	0.463							16	4	159
41 82 out	PBMC Proton Leak Respiration	BMIZ	0.384	0.581749	(-0.783, 1.551)	0.512							16	5	160

EST is the estimated linear effect (i.e., slope) of the VARIABLE. EST_SE is the standard error of the EST. CI95 is the 95% confidence interval for the linear effect (i.e., slope) of the VARIABLE. FVALUE is the F-statistic testing whether full model is different from 0. NumDF is the numerator degrees of freedome for FVALUE. DenDF is the denominator degress of freedom for FVALUE. PVALUE is the p-value testing whether the linear effect (i.e., slope) is 0. RMSE is the root mean square error from the regression model; this is the standard deviation of DEPENDENT after adjusting for VARIABLEs. RSQ is the R-square value of the regression model. MODEL_P is the p-value testing whether the regression model explains any variabilitiy in DEPENDENT.

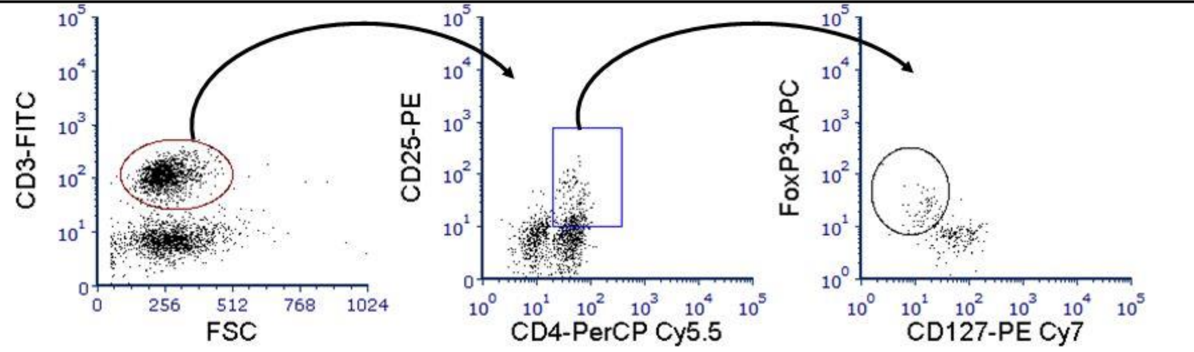

Figure 1: Gating Strategy to Define Tregs. PBMC were stained and gated based on CD3, CD4, CD25, CD127 and FoxP3 expression.
